# Effect of Aerobic Exercise on Serum Concentration of Apelin, TNFα and Insulin in Obese Women

**Published:** 2012

**Authors:** Shahin Sheibani, Parichehr Hanachi, Mohammad Ali Refahiat

**Affiliations:** 1 *Departmen of Exercise Physiology, Science and Research Branch, Islamic Azad University, Fars, Iran*; 2 *Faculty of Sciences, Biology Department, Biochemistry Unit, Alzahra University, Tehran, Iran*; 3 *Departmen of Exercise Physiology, Science and Research Branch, Islamic Azad University, Fars, Iran*

**Keywords:** Aerobic exercise, Apelin, Insulin, Obese women, TNFα

## Abstract

**Objective(s):**

Apelin is novel adipokine acting on APJ receptor, regulated by insulin and tumor necrosis factor-alpha (TNF-alpha). Plasma apelin levels are increased in obese subjects. The aim of this study was to investigate whether or not the aerobic exercise modifies the elevated plasma apelin levels in obese women.

**Materials and Methods:**

Twenty obese women (BMI 32.2 ± 6.4 kg/m^2^) were selected by random sampling method among obese women. Twelve healthy women with a BMI of 31.7 ± 0.6 kg/m^2^ served as control group. The aerobic exercise was performed during 8 weeks, 3 sessions per week. The intensity of the training program proceeded form 50% to 70% in 8 weeks.

**Results:**

Results showed that plasma levels of apelin and TNFα were higher in obese individuals compared with the control group. The exercise resulted in significant decrease (*P<*0.05) of BMI to 29.8± 6.3 kg/m^2 ^, plasma insulin (8.16 ± 0.73 to 6.58 ± 0.66 µmol/l), apelin (369 ± 25 pg/ml vs 257 ± 12 pg/ml) and TNFα levels (0.66 ± 0.04 pg/ml vs 0.56 ± 0.04 pg/ml*).* *P*< 0.05 was considered statistically significant.

**Conclusion:**

Exercise can decrease plasma apelin, insulin and TNFα levels in obese women. Regular physical activity causes a decrease in plasma levels of apelin if body mass index and body fat mass simultaneously decreased.

## Introduction

During the last decade, a growing number of adipocyt derived hormones or adipokines have been identified including leptin, adiponectin, and resistin (1). These adipokines have been described to be involved in physiological regulations of fat storage and development, metabolism, eating behavior plus playimg a role in obesity-associated disorders including type 2 diabetes and hypertension. Apelin is a novel adipokine acting on APJ receptor, regulated by insulin and tumor necrosis factor-alpha (TNF-alpha). Although apelin has been shown to be expressed in white adipose tissue (2), it is produced and secreted by human and mouse mature adipocytes (3, 4). Apelin is a bioactive peptide identified as the endogenous ligand of APJ, a G protein-coupled receptor (3, 5). Apelin peptides are derived from a 77- amino-acid precursor, which is processed to several active molecular forms such as apelin-36 or apelin-13 in different tissues and in the bloodstream (5). Apelin and its receptor APJ have been expressed in the hypothalamus, stomach, endothelial cells, vascular smooth muscle cells, cardiomyocytes in mouse adipocytes (6) and human osteoblasts (7). The most documented functions of apelin/APJ concern the regulation of fluid homeostasis (8) and the modifications of cardiac contractility and blood pressure . So far, little data is available regarding the regulation of apelin especially in humans. Daviaud *et al* (2006) reported that there is a strong correlation between apelin and TNFα expression in adipose tissue of lean and obese humans (6). Regulation of apelin expression by insulin (10) and TNFα (11) in human adipocytes or adipose tissue (AT) has also been reported. Moreover, the basal plasma levels of apelin are significantly higher in obese people compared to control lean individuals (10), correlating positively with body mass index (BMI) (12, 13). Also spontaneously hypertensive obese rats underwent swimming training consisting of 54 swimming sessions of 60 min each (6 days/week for 9 weeks). Findings showed that long-term swimming training with decreasing obesity relieved the pathogenesis of hypertension (14). However, findings in obese rats which underwent 2 hr swimming in one session showed that apelin and PGC-_1__α_ increased and consequently, basal rate of metabolism increased (15). These data suggest that apelin may play an important role in obesity. Therefore, the aim of this study was to investigate whether or not the increased plasma apelin levels previously reported in obese subjects would be reduced after 8 weeks of aerobic exercise in obese women. 

## Materials and Methods

***Subjects***

Thirty two women participated in this study, who were divided into two groups of experiment (n=20) and control (n=12). Twenty obese women (age: 38.6 ± 2.5 years, weight 72.95±1.2 kg , BMI of 32.2 ± 1.4 kg/m^2^ and fat mass 42.3± 1.1 %) participated as experiment group. To obtain references values, n=12 healthy and obese women (age 39.7 ± 2.2 years, weight 73.2 ± 1.3 kg, BMI 32.7 ± 1.6 kg/m^2^, fat mass 42.5± 1.9 %) were chosen as control group. Informed consent to participate in the study was obtained from each subject before the beginning of the experiments. All women were pre-menstruation, drug-free and, based on their medical history, clinical findings and entry laboratory examination, did not suffer from any disease but obesity. Their body weight was stable for at least three months before the beginning of the study. 

***Examination procedures***

The subjects fasting blood samples were taken between 9-10 in the morning. Plasma apelin, insulin and TNF alpha levels were assayed in 2 phases before exercise and 24 hr after the end of the eighth weeks exercise.

Body composition was assessed using Bodystat, Quad scan 4000, Isle of Man. The coefficients of variation of fat mass, fat free mass and impedance were 1.7%, 0.8% and 1.5% respectively. Blood samples were obtained from antecubital vein before exrcise and 24 hr after training and collected in test tubes containing EDTA and processed immediately in a refrigerated centrifuge. The plasma was stored at −80°C until biochemical analysis. 

**Table 1:** Clinical characteristics and metabolic parameters of controls and experimental subjects

	Obese-exercise	Obese-exercise	*P*	Obese-control	Obese-control	*P*
	Before exercise	After exercise		Before	After	
Weight(kg)	72.9±1.2	83.1±3.6	<0.0001	73.2 ± 1.3	73.9±1.1	>0.064
BMI(kg/m^2^)	32.2±1.4	29.8±1.4	<0.0001	32.7 ± 1.6	31.5±1.8	>0.069
Fat mass (%)	42.3±1.1	39.0±1.4	<0.001	42.5± 1.9	41.9±1.7	>0.058
Fat (kg)	37.8±2.6	33.3±2.7	<0.0001	37.5±2.2	36.9±2.0	>0.072

The data presented are means ± SEM in obese subjects in control (n=12), experiment (n=20) groups before and after aerobic exercise

***Food intake***

The diet was designed to provide 400 kcal/d less than the individually estimated energy requirement based on calculated resting metabolic rate multiplied by a coefficient of correction. The target macronutrient composition of the diet was 25-30 % of total energy from fat, 15 % protein and 55-60% from carbohydrate.

***Training protocol***

The experimental groups participated in aerobic exercise. Training procedure was supervised by an exercise mentor. The training program for aerobic exercise was performed during 8 weeks, 3 sessions each week in stadium of . The intensity of the training program proceeded from 50% to 55% (in the first 2 weeks), 55% to 60% (in the second 2 weeks), 60% to 65% (in the third 2 weeks) and 65% to 70% of maximum heart rate (in the last 2 weeks). The duration of training programs without the warm up and cool-down was 15 min. The intensity of training program was controlled and regulated. All subjects performed a warm up (20 min) and a cool-down (15 min) program in every training session. Before the beginning of the research, the subjects became familiar with the training procedure.

***Analytical methods***

Plasma insulin concentration was measured by RIA (). Plasma apelin levels were measured with a commercially available enzyme-linked immunoassay (ELISA) kit (Phoenix Pharm, ). The sensitivity of the assay was 0.2 ng/ml and the inter-assay error was below 5%. The ELISA had 100% cross-reactivity with human apelin-12, apelin13 and apelin-36. Concentrations of TNFα in plasma were measured using ultra-sensitive ELISA kit (Biosource International, USA).

***Statistical analysis***

Statistical analysis was performed using SPSS ver, 12.0 for Windows. Statistical analysis was done by parametric Wilcoxon’s test for paired observations. Correlations were analyzed by Spearman’s parametric test .Data were presented as means ± SEM. and *P* value<0.05 was considered significant. 

## Results

***Effect of aerobic exercise on metabolic variables***

The clinical parameters of the subjects are shown in Table 1. The aerobic exercise resulted in mean body weight loss of 6.7 ± 0.6 kg (7.4% loss of the initial body weight) without any changes in blood pressure. Moreover, an increase in insulin sensitivity of the subjects was observed (Table 1).

***Effect of aerobic exercise on plasma apelin, insulin and TNFα levels***

**Figure 1:**
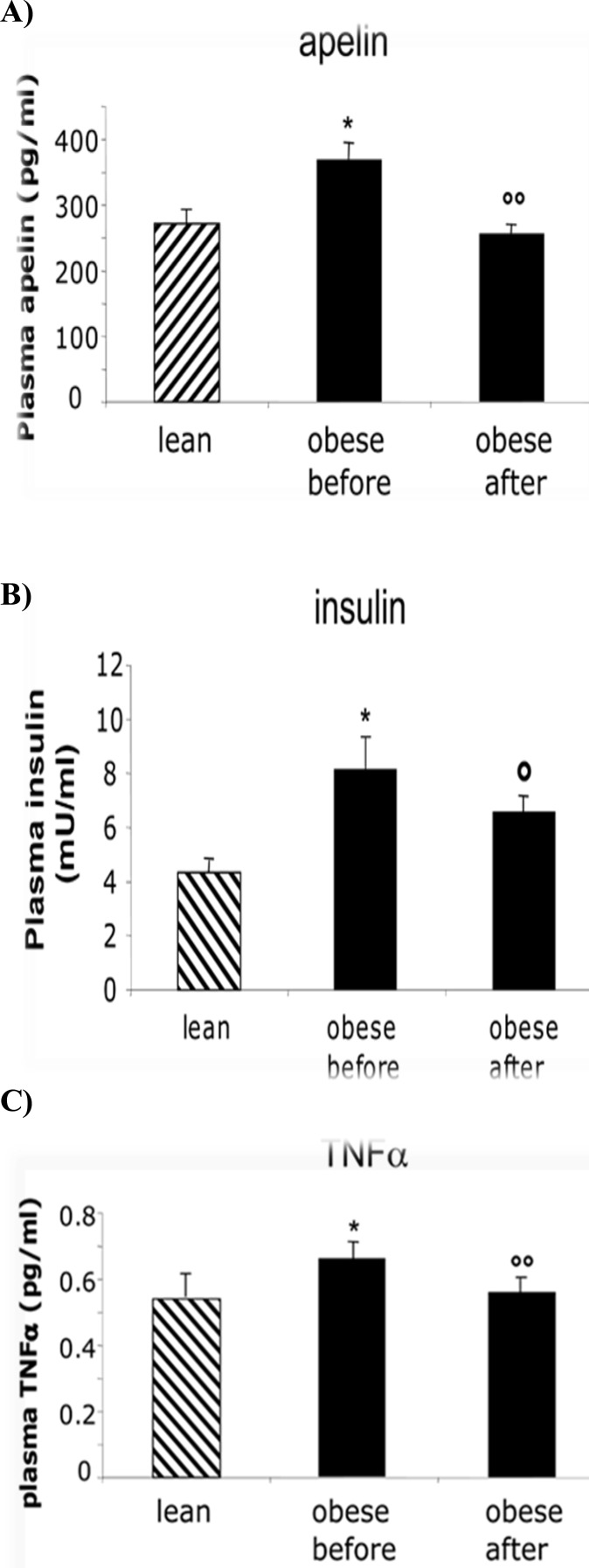
(A): Plasma apelin (pg/ml), (B): TNFα and (C): insulin and levels in control (n=12) and experimental groups before and after aerobic exercise (n= 20). Data are presented as mean ± SEM. * *P<* 0.05 compared with control subjects, °°*P* < 0.001 compared with experimental group before aerobic exercise. °*P<* 0.05 compared with experimental group before aerobic exercise.

Plasma levels of apelin were elevated in experiment group (369 ± 25 pg/ml, n=20) compared to control group (272 ± 20 pg/ml). After aerobic exercise, plasma levels of apelin in experiment group were significantly (*P<*0.05) decreased (Figure 1, A). The plasma concentrations of TNFα (0.66 ± 0.04 pg/ml, vs 0.54 ± 0.07 pg/ml,) and insulin (8.16 ± 0.73 (*P<*0.05) decreased (Figure 1, B&C).

The plasma concentrations of TNFα (0.66 ± 0.04 pg/ml, vs 0.54 ± 0.07 pg/ml,) and insulin (8.16 ± 0.73 mU/ml, vs 4.31± 0.56 mU/ml) were also higher in obese compared to control subjects, however, apelin, significantly (*P<*0.05) decreased after aerobic exercise (Figure 1).

***Associations of plasma apelin levels with metabolic variables***

The exercise-induced changes in plasma apelin levels directly correlated with the reduction of metabolic variables such as plasma insulin and TNFα levels (Table 1).

## Discussion

An increasing number of peptides are reported to be produced in the adipocyte. However, few of them have been shown to possess true endocrine potencies or be regulated during metabolic perturbations and changes in nutritional status. Many studies have causally associated these adipokines with a large panel of diseases such as type 2 diabetes or obesity (16). Elevated plasma apelin has been described by different research groups (10, 12) in severe obese. In present study we have shown that plasma apelin levels were increased in obese and positively correlated with BMI and fasting plasma insulin, This finding is similar to Boucher *et al *(2005), suggesting a role of apelin in the pathogenesis of obesity (11). Our assay and the assay used by Foldes in obese, shows that plasma insulin levels also significantly increased, suggesting that the regulation of apelin by insulin could occur in humans. However, no data were available on a potential reversal of the elevated plasma apelin in obese subjects. In the present study, the aerobic exercise was associated with weight and plasma apelin reduction levels. At the end of the exercise, the plasma apelin levels in experiment group approached nearly to the range in controls. It is interesting to note that the relationship between apelin and insulin or TNFα during obesity and obesity-associated disorders was still maintained at the end of the aerobic exercise but was dependent on the magnitude of insulin sensitivity (17). These finding corresponded to the result of Boucher *et al*, 2005 (11), Heinonen *et al*, 2005 (13) and Li *et al* 2006 (18), however, it was different from Wei *et al*, 2005 (19), Therefore, in agreement with previous studies, insulin and TNFα could be potential candidates involved in the regulation of decrease of apelin blood levels. The relationship of apelin to blood pressure changes was assessed, also correlation between change of plasma apelin and systolic blood pressure were determined. Thus, plasma apelin level decreased after aerobic exercise suggesting that the reduced in BMI could contribute to decreased circulating apelin levels. The present study demonstrates that, in obese women, the aerobic exercise associated with weight reduction and with a decrease of insulin resistance promotes a reduction of the elevated plasma apelin levels, although apelin has been viewed as a beneficial adipokine up-regulated in obesity (19). 

## Conclusions

In conclusion we investigated that, in the obese-exercise group, both plasma apelin and insulin levels were significantly higher, indicating that apelin homeostasis was impaired in the obese state and suggesting that the rise in plasma insulin could promote an increase in blood concentrations of apelin. It remains to establish whether the increased levels of apelin observed in obesity were an attempt to overcome either insulin resistance or obesity-related cardiovascular diseases or another metabolic defect such as apelin resistance. Thus, understanding the contribution of such an adipokine in obesity-associated disorders appears to be of major importance.
